# Supplementary material for: Three cooperative mechanisms required for recovery after brain damage
Source: Sci Rep. 2019 Nov 1;9:15858. doi: 10.1038/s41598-019-50946-y (PMC6825173; doi:10.1038/s41598-019-50946-y)

Supplementary Information
Three cooperative mechanisms required for recovery after brain damage

D. Berger,[1] E. Varriale,[2] L. Michiels van Kessenich,[1] H. J. Herrmann,[3,4] and L. de Arcangelis[5,6,*]

[1] *Computational Physics for Engineering Materials, IfB, ETH Zürich, CH*
[2] *Physics Department, University of Naples Federico II, 80125 Naples, Italy*
[3] *PMMH, ESPCI, 7 quai St. Bernard, 75005 Paris, France*
[4] *Departamento de Fisica, Universidade Federal do Ceará, 60451-970 Fortaleza, Ceará, Brasil*
[5] *Dept. of Engineering, University of Campania "Luigi Vanvitelli", Aversa (CE), Italy*
[6] *INFN sez. Naples, Gr. Coll. Salerno, Italy*

(Dated: September 13, 2019)

Fig. S1 Configuration of a neural network with diffuse damage Network configuration ($N = 200$) where a 30% diffuse damage is implemented: The damaged neurons are red and unaffected neurons are grey. The connections of all damaged neurons are removed.

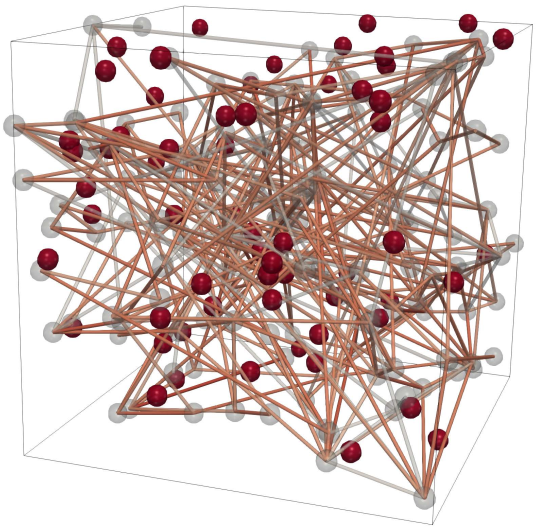

Fig. S2 Critical behaviour of avalanche activity Distribution of avalanche size for the system with $N = 10000$ neurons. Criticality depends on recovery rate: The avalanche size distribution for different values of the recovery rate Δu_r exhibits either a critical behaviour for $\Delta u_r = 7 \cdot 10^{-5}$ or a super / sub critical behaviour. The value of the recovery rate leading to criticality depends on the system size N.

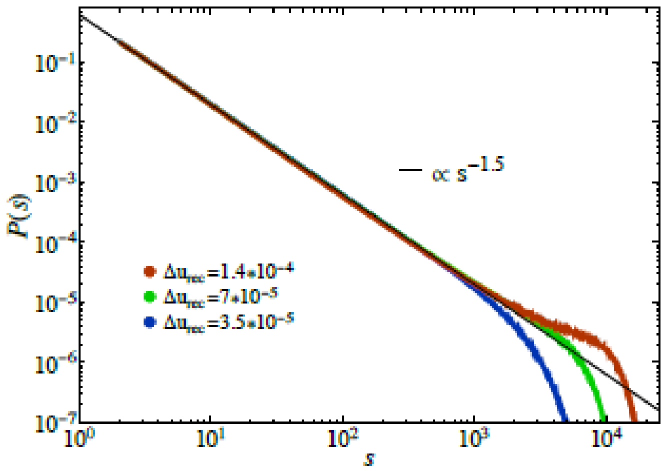

Fig.S3 Dependence on parameters of the recovery by plasticity reactivation. The recovery appears to be quite insensitive to, both, the number of plastic adaptations N_{rec} and the penumbra size.

Left: Dependence of the recovery on different extensions of plastic adaptation. Different curves correspond to localized damaged (ld 50%), undamaged (nd) and networks after plasticity reactivation for a penumbra size $20\% N$. (a) Rescaled avalanche size distribution $P(s)s^{1.5}$ as function of the rescaled size s/c. (b) Average firing rate f vs. s/c, labels as in (a). The inset shows the scatter plot of the average avalanche size $< s >$ vs. the cutoff c for the same color code.

Right: Dependence of the recovery on different penumbra sizes. Different curves correspond to damaged (ld 50%), undamaged (nd) and networks after plasticity reactivation for $N_{rec} = 20000$. (a) Rescaled avalanche size distribution $P(s)s^{1.5}$ as function of the rescaled size s/c. (b) Average firing rate f vs. s/c, labels as in (a). The inset shows the scatter plot of the average avalanche size $< s >$ vs. the cutoff c for the same color code.

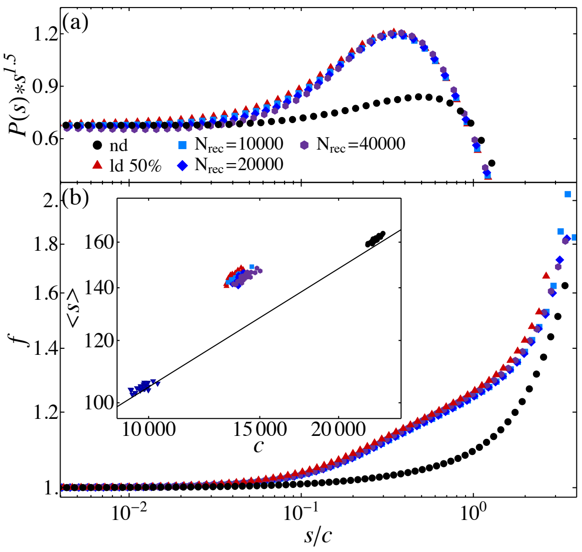

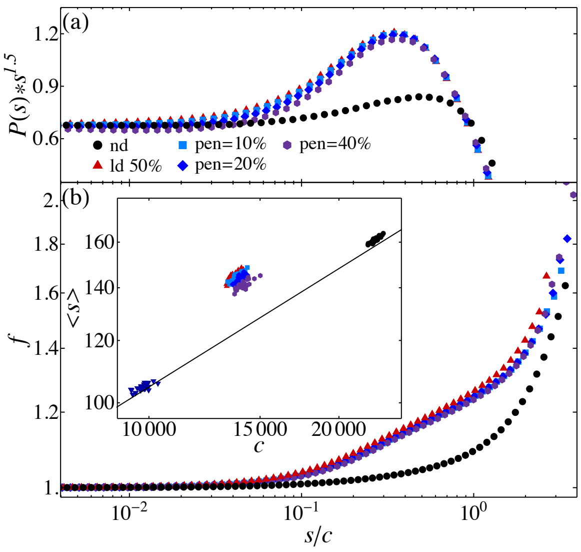

Fig.S4 Dependence on parameters of the recovery by hyperexcitability. The recovery improves with the variation of the firing threshold δt. However, for values larger than 0.2 an increase in the average firing rate is observed. Recovery also improves with the size of the penumbra.

Left: Dependence of recovery by hyperexcitability on different variations of the firing threshold δt. Different curves correspond to damaged (ld 50%), undamaged (nd) and networks after application of hyperexcitability for penumbra size 20%N. (a) Rescaled avalanche size distribution $P(s)s^{1.5}$ as function of the rescaled size s/c. (b) Average firing rate f vs. s/c, labels as in (a). The inset shows the scatter plot of the average avalanche size $< s >$ vs. the cutoff c for the same color code.

Right: Dependence of recovery by hyperexcitability on different penumbra sizes. Different curves correspond to damaged (ld 50%), undamaged (nd) and networks after application of hyperexcitability for $\delta t = 0.2$. (a) Rescaled avalanche size distribution $P(s)s^{1.5}$ as function of the rescaled size s/c. (b) Average firing rate f vs. s/c, labels as in (a). The inset shows the scatter plot of the average avalanche size $< s >$ vs. the cutoff c for the same color code.

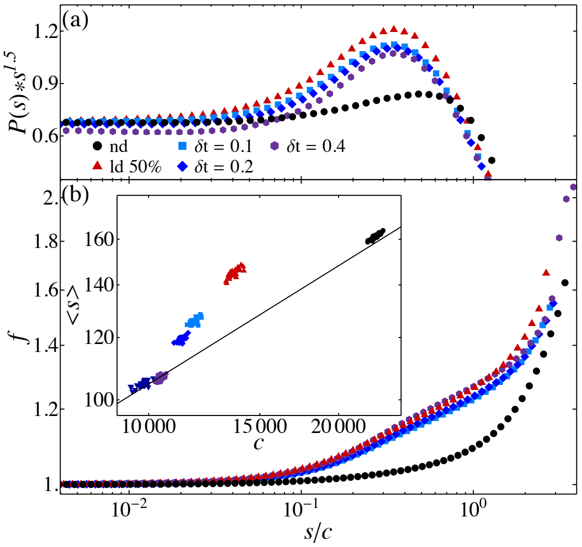

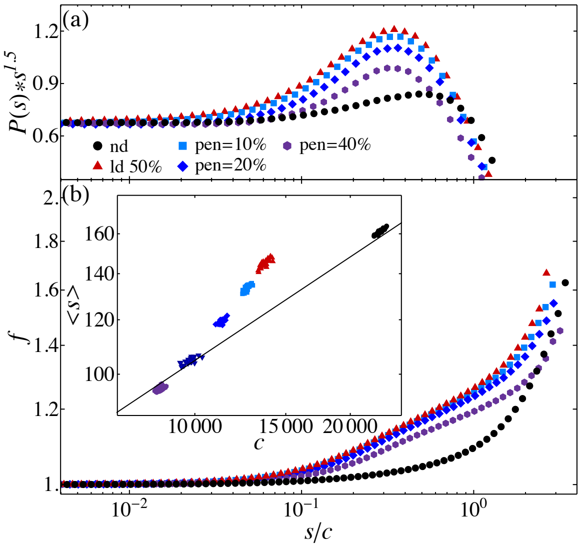

Fig.S5 Dependence on parameters of the recovery by synaptogenesis. The recovery improves with the number of sprouted synapses. Here the number is expressed in terms of the percentage of number of synapses killed by the stroke. For large values of newly sprouted synapses (10%) the system tends to become supercritical. Recovery improves with the average length of new synapses, r_0. This effect saturates and no appreciable difference is observed between data for $r_0 = 9$ and 12.

Left: Dependence of synaptogenesis on different percentage of newly sprouted synapses. Different curves correspond to damaged (ld 50%), undamaged (nd) and networks after creation of new synapses for $r_0 = 9$. (a) Rescaled avalanche size distribution $P(s)s^{1.5}$ as function of the rescaled size s/c. (b) Average firing rate f vs. s/c, labels as in (a). The inset shows the scatter plot of the average avalanche size $< s >$ vs. the cutoff c for the same color code.

Right: Dependence of synaptogenesis on different spatial extension of newly sprouted synapses r_0. Different curves correspond to damaged (ld 50%), undamaged (nd) and networks after creation of a number of new synapses equal to 5% the number of killed synapses. (a) Rescaled avalanche size distribution $P(s)s^{1.5}$ as function of the rescaled size s/c. (b) Average firing rate f vs. s/c, labels as in (a). The inset shows the scatter plot of the average avalanche size $< s >$ vs. the cutoff c for the same color code.

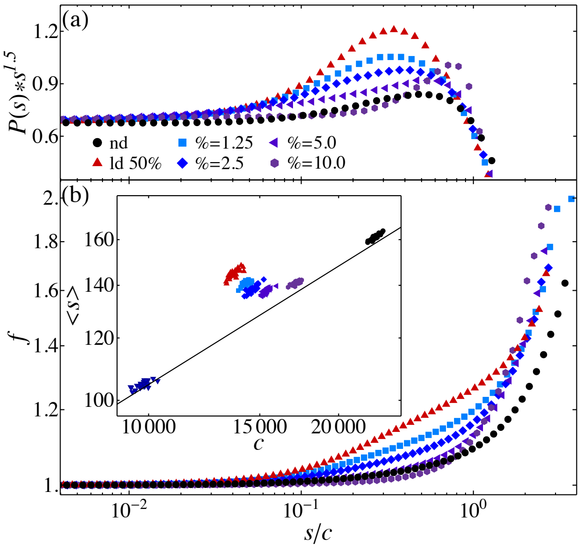

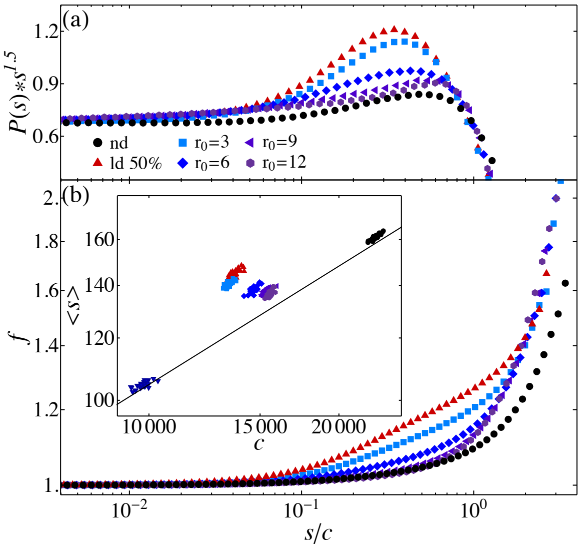

Fig.S6 Recovery after a diffuse damage. For diffuse damage only plasticity, with a limited effect, and synaptogenesis appear to help the recovery. Hyperexcitability has a negative effect, further reducing the cutoff and the average avalanche size. The three combined mechanisms bring the system even farther from the healthy state suggesting that the positive interplay between hyperexcitability and synaptogenesis does not occur in this case. The system size is $N = 10000$. (a) Rescaled avalanche size distribution $P(s)s^{1.5}$ as function of the rescaled size s/c for the damaged (dd 50%) and undamaged networks (nd), for the three recovery mechanisms applied separately and in a combined way. The parameters are: $N_{rec} = 20000$, $\delta t = 0.2$, $r_0 = 6$. (b) Average firing rate f vs. s/c for undamaged (nd), diffuse damage (dd 50%) and the three recovery mechanisms as in (a). The inset shows the scatter plot of the average avalanche size $< s >$ vs. the cutoff c for the same color code.

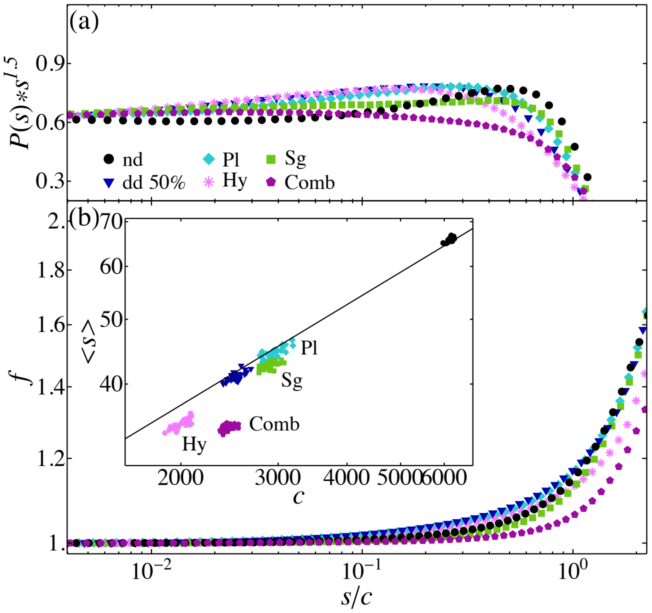

Supplement: Supplementary file 1 — Supplementary information [file 41598_2019_50946_MOESM1_ESM.pdf]
